# Role of NCF2 as a potential prognostic factor and immune infiltration indicator in hepatocellular carcinoma

**DOI:** 10.1002/cam4.5597

**Published:** 2023-01-20

**Authors:** Ning Huang, Jing Zhang, Shuwen Kuang, Zhuo Li, Hong Zhao, Jianxiong Wu, Mei Liu, Liming Wang

**Affiliations:** ^1^ Department of Hepatobiliary Surgery, National Cancer Center/National Clinical Research Center for Cancer/Cancer Hospital Chinese Academy of Medical Sciences and Peking Union Medical College Beijing China; ^2^ Department of Pathology, National Cancer Center/National Clinical Research Center for Cancer/Cancer Hospital Chinese Academy of Medical Sciences and Peking Union Medical College Beijing China; ^3^ Laboratory of Cell and Molecular Biology & State Key Laboratory of Molecular Oncology, National Cancer Center/National Clinical Research Center for Cancer/Cancer Hospital Chinese Academy of Medical Sciences and Peking Union Medical College Beijing China

**Keywords:** HCC, NCF2, prognosis, TCGA, TME

## Abstract

**Background:**

Hepatocellular carcinoma (HCC) is one of the major causes of cancer‐related deaths globally. The tumor microenvironment (TME) plays a crucial role in the prognosis and treatment of HCC. Hence, it is important to exploit new biomarkers for survival surveillance and TME estimation of HCC.

**Methods:**

HCC samples data was collected from The Cancer Genome Atlas (TCGA) and International Cancer Genome Consortium (ICGC) database, and clinical samples were collected from our center. The TME of HCC were explored with ESTIMATE (Estimation of STromal and Immune cells in MAlignant Tumor tissues using Expression data), ssGSEA (single sample Gene Sets Enrichment Analysis) and CIBERSORT algorithm. Differentially expressed genes were analyzed with functional enrichment analysis. Immunohistochemistry was implemented to validate the results.

**Results:**

Based on TCGA database, we found that Neutrophil Cytosolic Factor 2 (NCF2) was significantly associated with the prognosis of HCC patients, involved in immune‐related biological processes of HCC and closely associated with some types of immunocompetent cells. The survival analysis based on NCF2 expression assessed by immunohistochemistry also confirmed that NCF2‐positive group had a shorter relapse free survival (RFS) and overall survival (OS) than NCF2‐negative group. Multivariate Cox regression revealed NCF2 expression level and lymphovascular space invasion (LVSI) were independent risk factors for HCC patients. Receiver operating characteristic curves showed that the combination of NCF2 and LVSI had higher predictive efficacy on the 1‐year RFS rate and 5‐year OS rate than each of them alone. Besides, the expression level of NCF2 was positively associated with M0 and M2 macrophages infiltration. Furthermore, NCF2 expression was positively correlated with CSF1, IL4, IL10, CD206, CD163, CSF1R and TGFβ1.

**Conclusion:**

We proposed that higher NCF2 expression predicted an adverse prognosis and more M2 macrophages infiltration in HCC patients.

## Graphical abstract

Prognostic and immune related genes screened out in TCGA‐LIHC dataset were used for establishing a prognostic model. And a hub gene, NCF2, was identified as an independent prognostic factor of HCC patients as well as a potential factor to modulate the tumor microenvironment (TME) in HCC via regulating the polarization of macrophages.

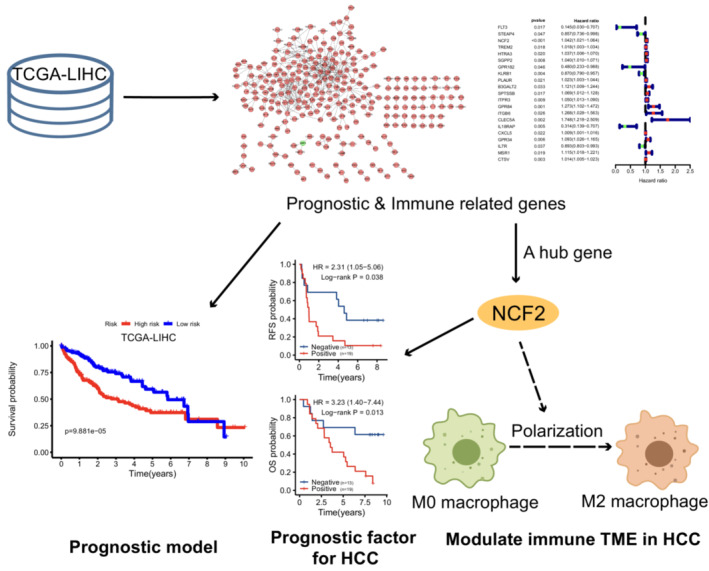

## 1 INTRODUCTION

Hepatocellular carcinoma (HCC) is the sixth most diagnosed malignant tumor and the third leading cause of cancer‐related death worldwide.^1^ Cirrhosis, resulting from virus infection, alcohol abuse, nonalcoholic fatty liver disease, etc., is the major risk factor of HCC. Usually the OS rate of HCC patients at locally advanced stage is less than 10% in 5 years.^2^ In addition, disease recurrence happens to 70% patients following curative‐intent therapy.^3^ Traditional risk factors for recurrence of HCC include tumor size, satellite nodules, microvascular invasion and poor tumor differentiation.^4^, ^5^, ^6^ And AFP is still thought highly in the evaluation of HCC recurrence and patients' survival. In spite of the remarkable therapeutic progression, the mortality of HCC patients is still high. Specific features and sensitive biomarkers facilitate the prediction of prognosis, and help doctors to adjust therapeutic regimen. Therefore, developing new and more reliable strategies for survival and recurrence surveillance is beneficial to clinical practice and disease outcome.

The tumor microenvironment (TME), comprising immune cells, stromal cells, blood vessels and extracellular matrix, is gradually revealed to have influence on the prognosis of cancer patients and continuously evolving for its interaction with cancer cells, including HCC. Natural killer (NK) cells contribute to innate immune response against virus and tumors, and they account for 30%~50% of intrahepatic lymphocytes.^7^ The abundance and infiltration of NK cells in peripheral blood and TME are correlated with the relapse and overall survival of HCC patients.^8^, ^9^ FoxP3 is widely regarded as a marker of regulatory T (Treg) cells, and it has been found independently associated with microvascular invasion, tumor size and envelope invasion in HCC, and significantly correlated with the recurrence of HCC.^10^ In addition, Tumor‐associated macrophages (TAMs) are arousing increasing focus from researchers. M2 macrophages were reported as an adverse predictive factor for HCC patients.^11^, ^12^ Immunity plays an important role in carcinogenesis and cancer progression, and finding out how it works matters a lot to the prognosis and therapy of cancer. However, little work has been done to study the relationship between the TME and cancer prognosis.

Recently, TCGA (https://cancergenome.nih.gov/), a database recording genomics, proteomics, mutation and clinical data of various tumor samples, has been frequently used by many researchers to exploit the unknown information contained in nucleic acid and protein. In addition, some practical algorithms like the ESTIMATE algorithm,^13^ ssGSEA algorithm^14^ and CIBERSORT algorithm^15^ are also universally used for the assessment of TME. Based on gene sets of specific cells, all these algorithms help to better present stromal and immune cells infiltration landscapes in tumor tissue. Therefore, making good and proper use of these bioinformatics technologies, we can find out the TME of HCC in greater detail, and get inspiration for further study.

Herein, we used transcriptome and clinical data from TCGA to screen out a novel prognosis‐related gene for HCC patients, and then explored its prognostic value by using the clinical tumor samples. Furthermore, we evaluated the immune cells infiltration in the TME of HCC and predicted its relationship with the prognosis‐related gene. Not only did our study reveal a novel biomarker for predicting the outcome of HCC patients, but also implied a potential mechanism that it might influence the progression of HCC by regulating the TME of HCC, which could eventually provide a new idea for clinical treatment.

## 2 MATERIALS AND METHODS

### 2.1 Assessment of the TME of HCC and DEGs Identification

RNA‐Seq and relevant clinical data of 424 HCC samples (TCGA cohort, 50 normal samples vs. 374 tumor samples) were obtained from TCGA in January, 2022. Another 242 HCC samples were downloaded from ICGC database (LIRI‐JP dataset). The infiltration of stromal cells and immune cells in the microenvironment of HCC and tumor purity of HCC were assessed with ESTIMATE algorithm, and measured by StromalScore, ImmuneScore and ESTIMATEScore, respectively. The relative abundance of tumor infiltrating immune cells (TIICs) were evaluated by ssGSEA^16^, ^17^ and CIBERSORT algorithm (perm = 1000, results with *p* < 0.05 were considered credible) with R (version 3.6.3).

Differentially expressed genes (DEGs) were screened out with “limma” package in R (version 3.6.3). The DEGs were determined at the absolute value of log2 fold change (|log2FC|) > 1 and the false discovery rate (FDR) < 0.05. The “VennDiagram” package was utilized to extract the genes which were expressed at similar level in both immune and stromal cells.

### 2.2 Functional analysis of DEGs

Gene Ontology (GO) and Kyoto Encyclopedia of Genes and Genomes (KEGG) analyses were executed with R (version 3.6.3) packages “ggplot2,” “enrichplot,” and “clusterProfiler”, which showed the biological processes, cellular components, molecular functions and pathways related to the DEGs. The statistical significance was considered as *q*‐value <0.05.

Gene Set Enrichment Analysis (GSEA) was implemented using GSEA software (version 4.1.0, Broad Institute, Cambridge, MA, United States) to testify the pathways associated with NCF2. NOM *p*‐value <0.05 and FDR <0.25 was thought significantly different.

To explore the interaction among DEGs, the protein–protein interactions (PPI), of whom the interactive confidence was greater than 0.95 on the STRING platform (version 11.5), were selected to establish an interaction network with Cytoscape software (version 3.8.2).

### 2.3 Prognostic model

The prognostic model was constructed with “survival” package in R (version 3.6.3). The genes included in this model was selected in the prognosis and immune related genes with function “step” in “survival” package, which can optimize the model. The prognostic model was represented by riskScore = ∑ gene expression (FPKM) × coef.

### 2.4 Clinical specimens and follow‐up

Thirty‐two HCC clinical samples (Table S1) from Hepatobiliary Surgery Department, Cancer Hospital Chinese Academy of Medical Sciences (CHCAMS cohort) were used in the present study. All HCC tissues received a histopathological diagnosis. The patients received operation during April, 2012 to December, 2013, and then experienced standard follow‐up carried out through hospital‐based follow‐up visit and/or phone call until January 10, 2022. The interval of postoperative follow‐up was 3 months for the first 2 years and thereafter 6 months. Each follow‐up visit included Alpha‐fetoprotein (AFP), liver function, enhanced computed tomography (CT) and digital subtraction angiography (DSA). Any suspicious recurrence of HCC was confirmed by enhanced magnetic resonance imaging (MRI). Relapse free survival (RFS) was defined as the time between operation and recurrence, and OS was defined as the time between operation and death or the last follow‐up visit.

### 2.5 Immunohistochemistry

Paraffin embedded HCC tissues of CHCAMS Cohort were used for immunohistochemistry (IHC). After deparaffinization and hydration, heat‐induced method was performed for antigen retrieval. Primary antibody of NCF2 (ab109366, 1:25, Abcam, USA) was incubated at 4°C overnight. Sections were washed with TBS‐T buffer, and then incubated with secondary antibody, and finally stained with DAB. Semiquantitative assessment of the stained sections were determined by the proportion of positive cells and the staining intensity: “−” = no positive cells; “+” = 0 ~ 25% and weak or moderate intensity; “++” = 25% ~ 50% and moderate intensity, or 0 ~ 25% and strong intensity, or 50% ~ 100% and weak intensity; “+++” = 50% ~ 100% and strong intensity. All the sections were scored independently by two experienced pathologists.

### 2.6 Statistical analysis

Data are presented as mean ± SEM. Cut‐offs were provided by X‐tile software (Yale School Of Medicine, USA, version 3.6.1).^18^ Survival analysis was conducted by Kaplan–Meier method and Log‐rank test, and patients without completed follow‐up data were excluded. Differences between two groups were compared with t test. The independent risk factor analysis was conducted with univariate and multivariate Cox regression analysis (backward LR), and patients without any one characteristic were excluded. The correlation between two variables was determined with spearman's correlation analysis. Statistically significant difference was set as *p* < 0.05. All statistical analyses were performed utilizing R (version 3.6.3) (Figure S1).

## 3 RESULTS

### 3.1 837 DEGs were identified based on the different stromal and immune infiltration

**FIGURE 1:**
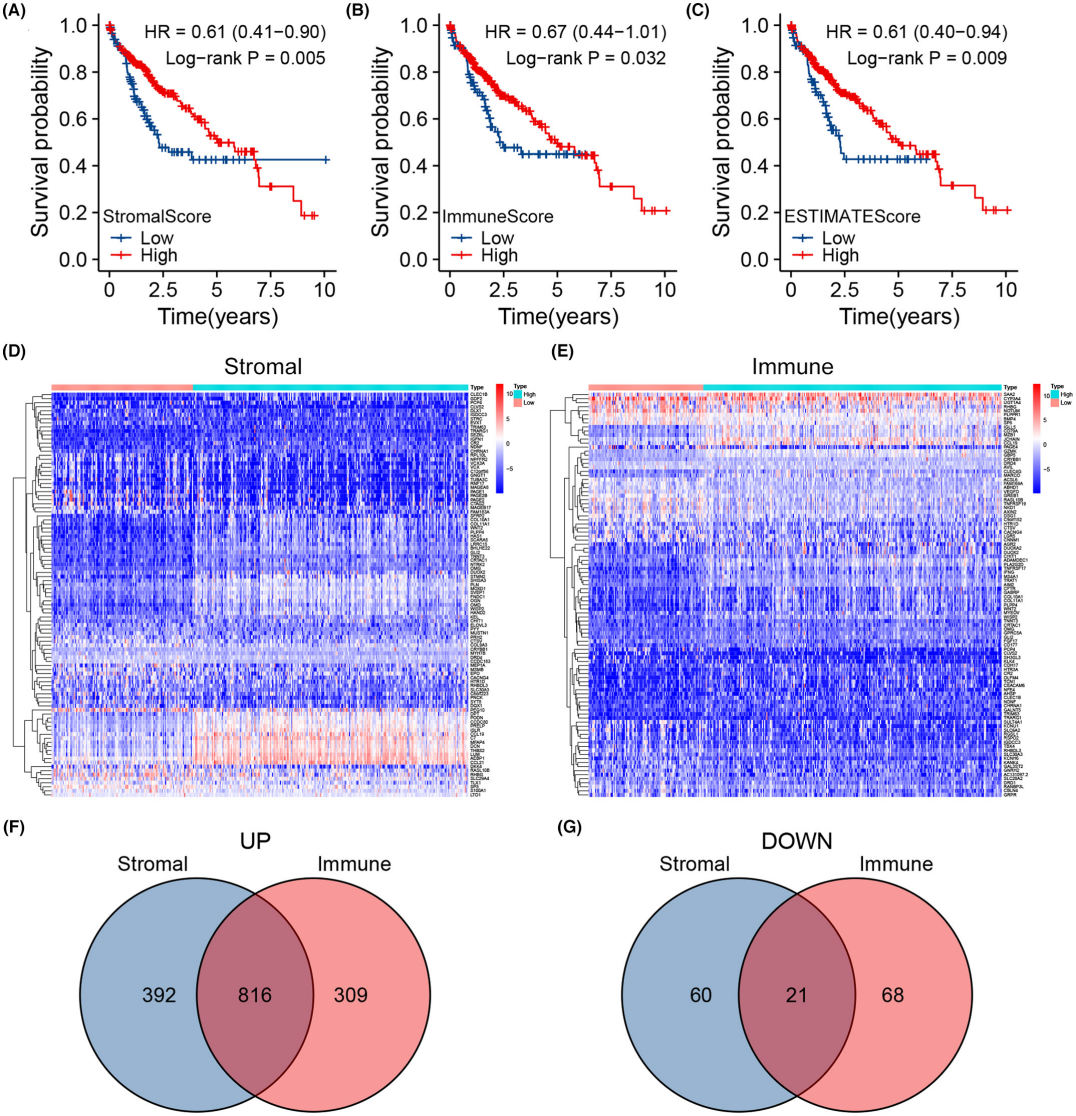
(A) Survival analysis of high‐ and low‐stromal‐score groups based on the cut‐off −922.6 (*n* = 371). (B) Survival analysis of high‐ and low‐immune‐score groups based on the cut‐off 23.82 (*n* = 371). (C) Survival analysis of high‐ and low‐estimate‐score groups based on the cut‐off −930.5 (*n* = 371). (D) A heatmap displaying the DEGs between high‐ and low‐stromal‐score groups. (E) A heatmap displaying the DEGs between high‐ and low‐immune‐score groups. (F) A venn diagram showing genes that were upregulated in high‐stromal and high‐immune score groups. (G) A venn diagram showing genes that were downregulated in high‐stromal and high‐immune score groups.

After analyzing the gene expression profile from TCGA database with ESTIMATE algorithm, StromalScore, ImmuneScore and ESTIMATEScore of each sample were obtained. For each score, we picked the best cut‐off (−922.6 for StromalScore, 34.0 for ImmuneScore and − 930.5 for ESTIMATEScore) to divided samples into high‐ and low‐score groups for survival analysis. And the results indicated that the prognoses of high‐StromalScore group, high‐ImmuneScore group and high‐ESTIMATEScore group were respectively better than that of the low‐StromalScore group, low‐ImmuneScore group and low‐ESTIMATEScore group (Figure 1A, *p* = 0.005, HR = 0.61, 95%CI 0.41–0.90; Figure 1B, *p* = 0.032, HR = 0.67, 95%CI 0.44–1.01; Figure 1C, *p* = 0.009, HR = 0.61, 95%CI = 0.40–0.94).

Since the prognosis was significantly different between high‐ and low‐score groups, we screened out the DEGs between groups. There were 1208 genes upregulated and 81 genes downregulated in high‐StromalScore group (Figure 1D,F,G), and 1125 genes upregulated and 89 genes downregulated in high‐ImmuneScore group (Figure 1 E–G). DEGs referred to genes commonly upregulated and downregulated in both two groups, and totaled 837 genes (Figure 1F,G).

### 3.2 ITGB6, NCF2 and PLAUR were identified as TME and prognosis‐related genes for HCC patients

**FIGURE 2:**
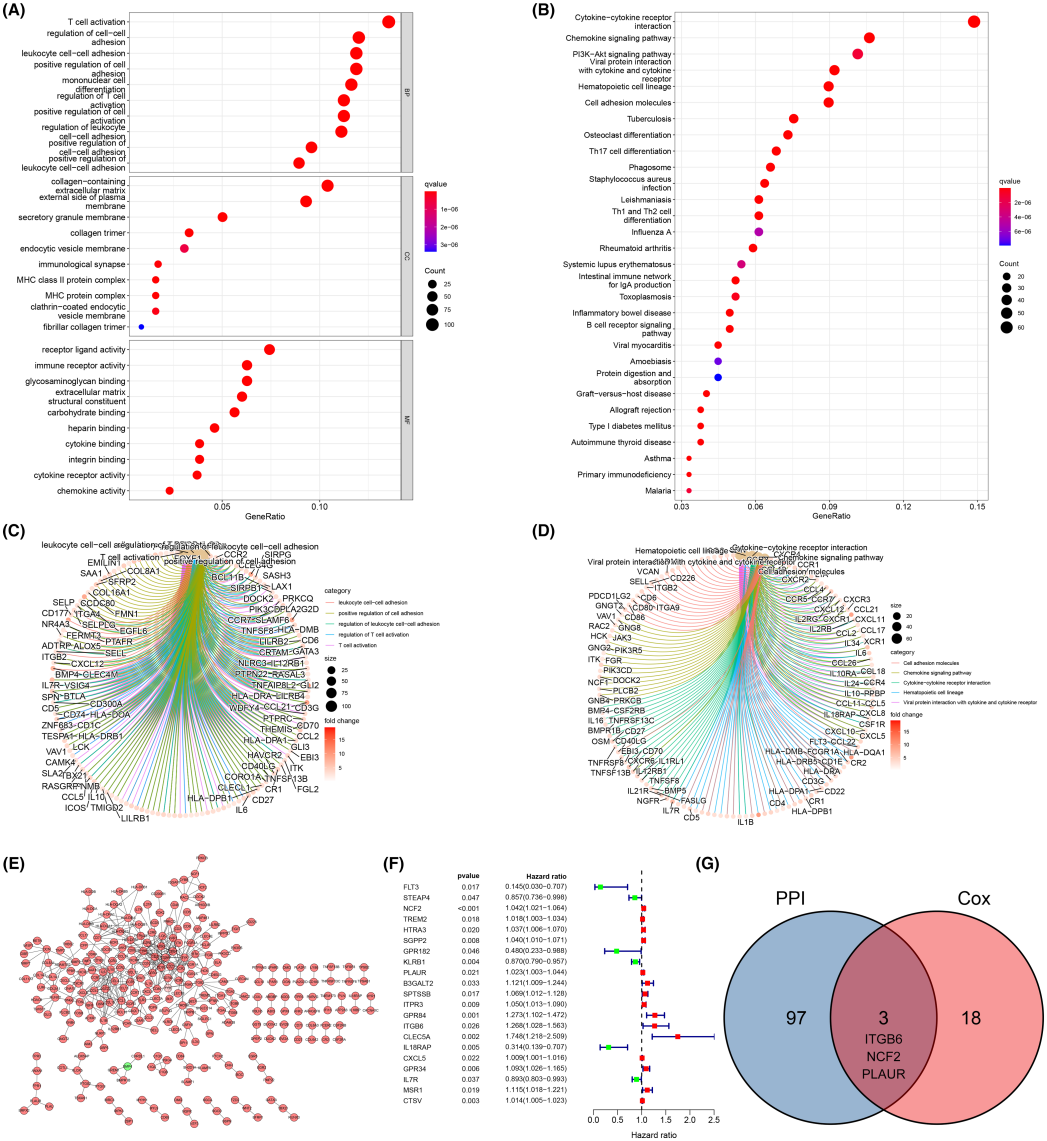
(A) Bubble plot and (C) circle plot of GO analysis, showing the molecular functions of DEGs. (B) Bubble plot and (D) circle plot of KEGG analysis, showing the relevant pathways of DEGs. (E) PPI network showing the interaction of DEGs. (F) Univariate Cox regression analysis of DEGs (*n* = 371). (G) Venn diagram providing three common genes (ITGB6, NCF2, PLAUR) of the results of PPI and Cox regression analysis.

In order to explore the functions of DEGs, and figure out whether they were related to immune, we performed functional enrichment analysis. GO analysis showed the DEGs‐associated biological processes, and KEGG analysis demonstrated the pathways in which these DEGs participated. Most DEGs function in cell adhesion, T cell activation, mononuclear cell differentiation, etc. (Figure 2A), and they engage in many immune‐related pathways, such as cytokine‐cytokine receptor interaction, chemokine signaling pathway, PI3k‐Akt pathway and hematopoietic cell lineage (Figure 2B). Figure 2C,D respectively shows the top 5 biological functions and pathways in which most DEGs were enriched.

To investigate the interactive relationship among DEGs, we built the PPI network (Figure 2E), and found 21 prognosis‐related genes by employing univariate Cox regression (Figure 2F). Then we exerted an intersection analysis between the top 100 PPI hub genes and the 21 prognostic genes, and found three intersection genes, ITGB6, NCF2 and PLAUR (Figure 2G), which meant they could have an active effect on the TME of HCC and the prognosis of HCC patients.

**FIGURE 3:**
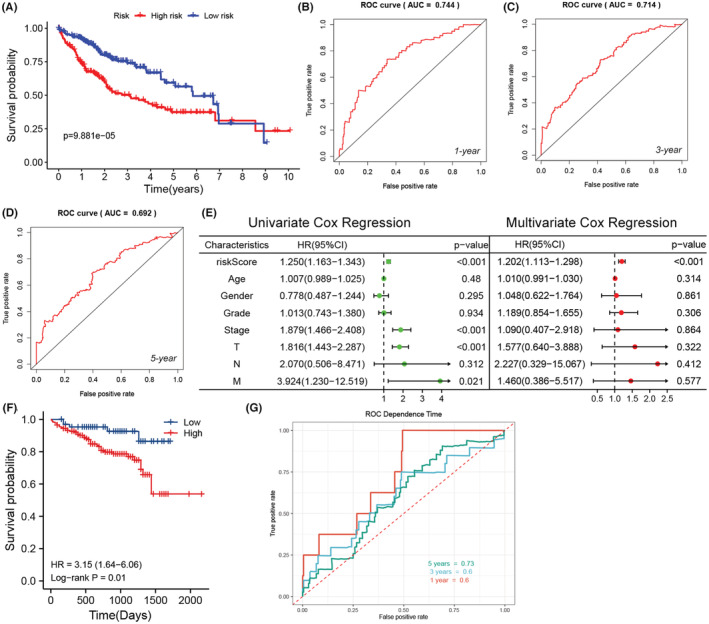
(A) Survival analysis based on the riskScore (*n* = 371). (B–D) 1‐year, 3‐year, 5‐year ROC of the prognostic model. (E) Univariate and multivariate Cox Regression analysis of riskScore and other characteristics (*n* = 235). (F) The survival analysis of LIRI‐JP dataset in ICGC database based on the prognostic model. (G) The ROC of LIRI‐JP dataset in ICGC database based on the prognostic model.

In addition, we constructed a prognostic model with the prognosis and immune related genes mentioned above. The genes included in this model and the associated parameters like coefficient, HR, HR95%CI were shown in Figure S2A. All samples were divided into high risk and low risk group by the median value of riskScore (Figure S2B). As expected, high risk group showed poor prognosis compared with low risk group (Figure S2C). And the expression discrepancy of these genes between high and low risk group were consistent with the coefficient (Figure S2D). In order to appraise the efficacy of this model, we performed survival analysis, ROC curve analysis and Cox regression analysis. It turned out that high risk group had worse prognosis (Figure 3A). Furthermore, this model did well in predicting 1‐year (Figure 3B, AUC = 0.744), 3‐year (Figure 3C, AUC = 0.714) and 5‐year (Figure 3D, AUC = 0.692) survival. The Cox regression analysis showed that the riskScore was an independent prognostic factor (Figure 3E). When we applied this model to LIRI‐JP dataset, we found it excellent (Figure 3F,G).

### 3.3 NCF2 acts as an independent prognostic factor in HCC patients

**FIGURE 4:**
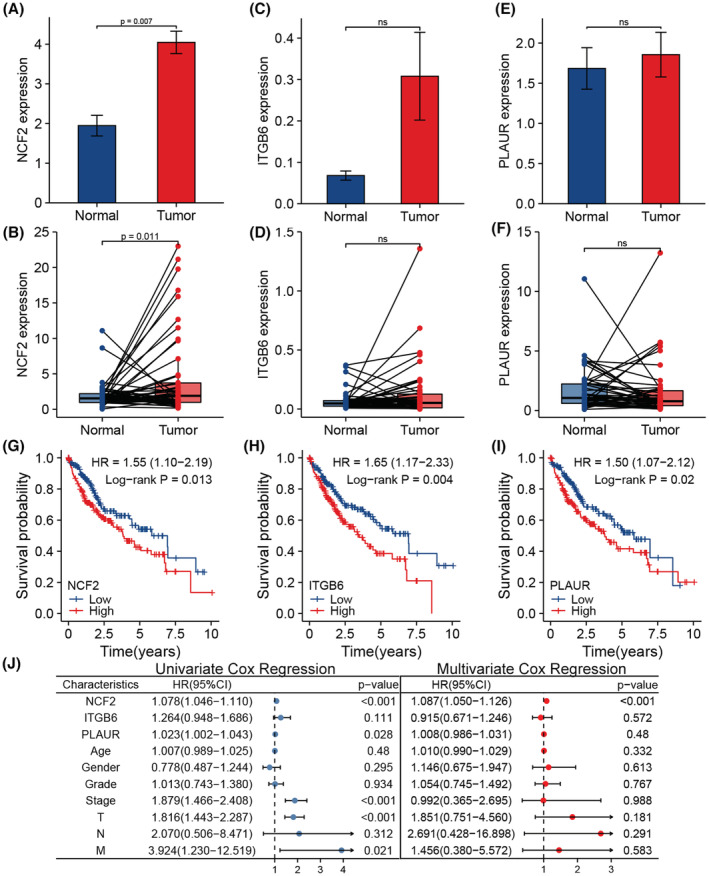
(A) Differential expression of NCF2 between unpaired tumor and normal tissue. (B) Differential expression of NCF2 between paired tumor and normal tissue. (C) Differential expression of ITGB6 between unpaired tumor and normal tissue. (D) Differential expression of ITGB6 between paired tumor and normal tissue. (E) Differential expression of PLAUR between unpaired tumor and normal tissue. (F) Differential expression of PLAUR between paired tumor and normal tissue. (G) Result of survival analysis based on NCF2 expression level of the TCGA cohort (*n* = 371). (H) Result of survival analysis based on ITGB6 expression level of the TCGA cohort (*n* = 371). (I) Result of survival analysis based on PLAUR expression level of the TCGA cohort (*n* = 371). (J) Result of univariate and multivariate Cox regression analysis of the TCGA cohort (*n* = 235).

Gene expression difference analysis manifested that NCF2 was differentially expressed in normal and tumor tissue, rather than ITGB6 and PLAUR (Figure 4A–F). Next, we dissected the prognostic value of these three genes mentioned above. We divided the TCGA cohort into 2 groups with the median expression value of these genes as a threshold, and performed survival analysis. It turned out that higher expression level of NCF2, ITGB6 and PLAUR predicted a poor prognosis (Figure 4G, *p* = 0.013, HR = 1.55, 95%CI 1.10–2.19; Figure 4H, *p* = 0.004, HR = 1.65, 95%CI 1.17–2.33; Figure 4I, *p* = 0.02, HR = 1.50, 95%CI = 1.07–2.12).

Then we further explore the prognostic value of these three genes. Univariate and Multivariate Cox regression analysis showed that NCF2 expression level could be an independent risk factor for HCC patients (Figure 4J, *p* < 0.001, HR = 1.087, 95%CI = 1.050–1.126). Whereas the expression level of ITGB6 and PLAUR were not independent risk factors, for their prognostic value was not significant when combined with clinicopathological characteristics like age, gender, grade, stage, T, N and M classification (Figure 4J, *p* = 0.572, HR = 0.915, 95%CI = 0.671–1.246; *p* = 0.48, HR = 1.008, 95%CI = 0.986–1.031).

### 3.4 IHC analysis confirmed the prognostic role of NCF2 in HCC patients

**FIGURE 5:**
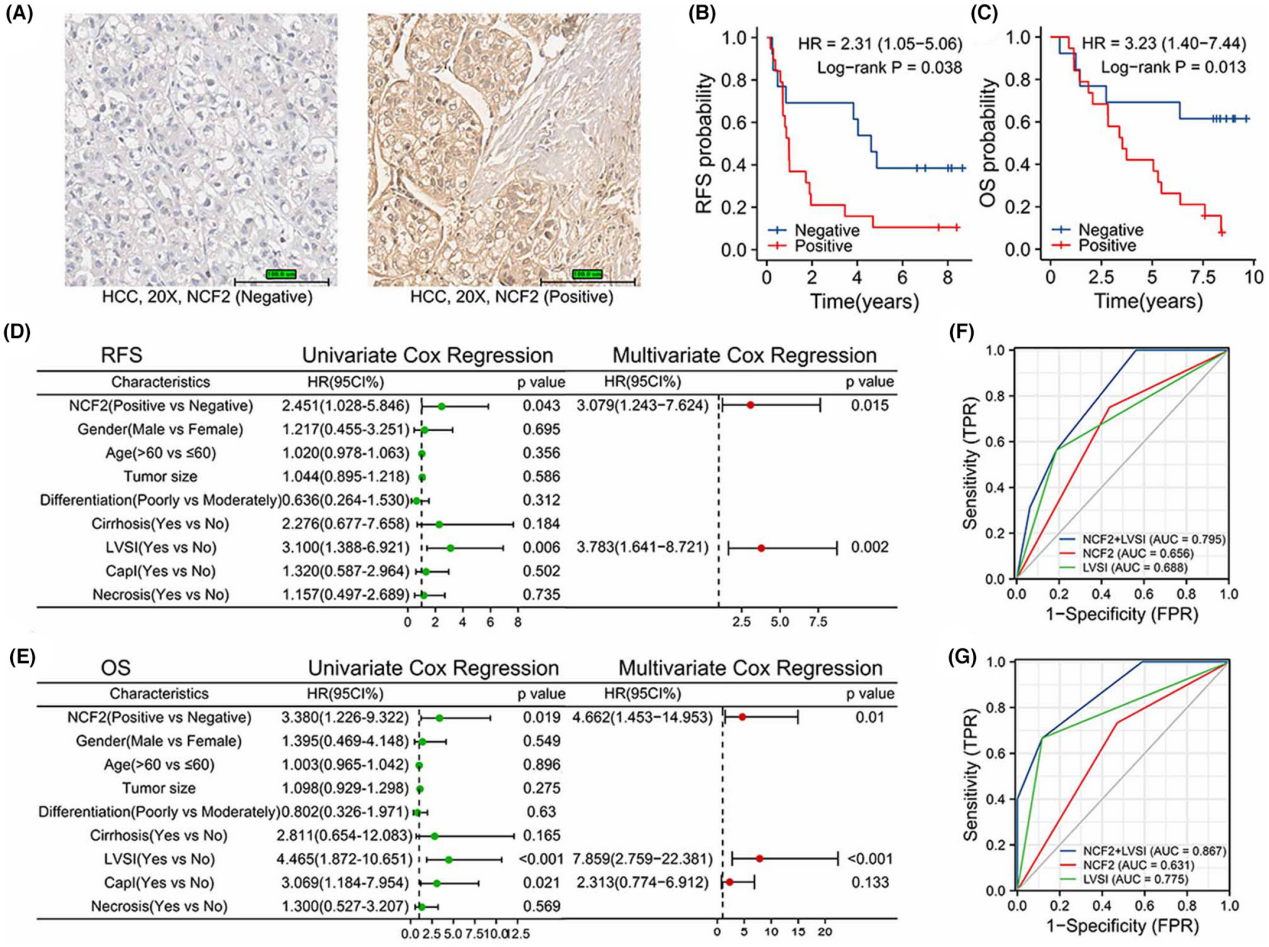
(A) Stained sections of Immunohistochemistry. (B) Relapse free survival analysis of the CHCAMS cohort based on IHC of NCF2. (C) Overall survival analysis of the CHCAMS cohort based on IHC of NCF2. (D) Result of Cox regression analysis of relapse free survival of the CHCAMS cohort. (E) Result of Cox regression analysis of overall survival of the CHCAMS cohort. (F) A ROC curve showing the predictive efficacy of NCF2, LVSI and their combination on 1‐year RFS rate. (G) A ROC curve showing the predictive efficacy of NCF2, LVSI and their combination on 5‐year OS rate.

To further prove the prognostic role of NCF2 in HCC patients, we detected its expression at protein level with IHC. According to the result of semiquantitative assessment of the stained sections, the CHCAMS cohort was divided into 2 groups. 13 samples were marked “−”, and were defined into NCF2‐negative group, while 19 samples, marked “+” or “++”, were defined into NCF2‐positive group (Figure 5A).

With the follow‐up data, we performed survival analysis, which validated that NCF2 was a prognostic factor for both RFS and OS of HCC patients. It turned out that NCF2‐negative group had higher RFS rate (Figure 5B, *p* = 0.038, HR = 2.31, 95%CI = 1.05–5.06) and OS rate (Figure 5C, *p* = 0.013, HR = 3.23, 95%CI = 1.40–7.44) than NCF2‐positive group. Then, we implemented univariate Cox regression analysis, combining NCF2 expression level with some clinicopathological information we had got, such as age, gender, tumor size, differentiation, cirrhosis, lymphovascular space invasion (LVSI), capsular invasion (CapI), and necrosis. The factors with *p* < 0.1 were enrolled in multivariate Cox regression analysis. Interestingly, the results showed that NCF2 expression level and LVSI were both independent prognostic factors for the OS and RFS of HCC patients (Figure 5D, *p* = 0.015, HR = 3.079, 95%CI 1.243–7.624; *p* = 0.002, HR = 3.783, 95%CI 1.641–8.721; Figure 5E, *p* = 0.01, HR = 4.662, 95%CI 1.453–14.953; *p* < 0.001, HR = 7.859, 95%CI 2.759–22.381). And these results were consistent with what we had discovered from the TCGA cohort.

Next, we compared the predictive efficacy of NCF2, LVSI and the combination of them on RFS and OS. The receiver operating characteristic (ROC) curve showed that the combination had higher predictive efficacy for 1‐year RFS rate (Figure 5F, AUC of NCF2 + LVSI vs. NCF2 vs. LVSI = 0.795 vs 0.656 vs. 0.688) and 5‐yeas OS rate (Figure 5G, AUC of NCF2 + LVSI vs. NCF2 vs. LVSI = 0.867 vs. 0.631 vs 0.775).

### 3.5 NCF2 could influence the infiltration of macrophages in the TME of HCC

**FIGURE 6:**
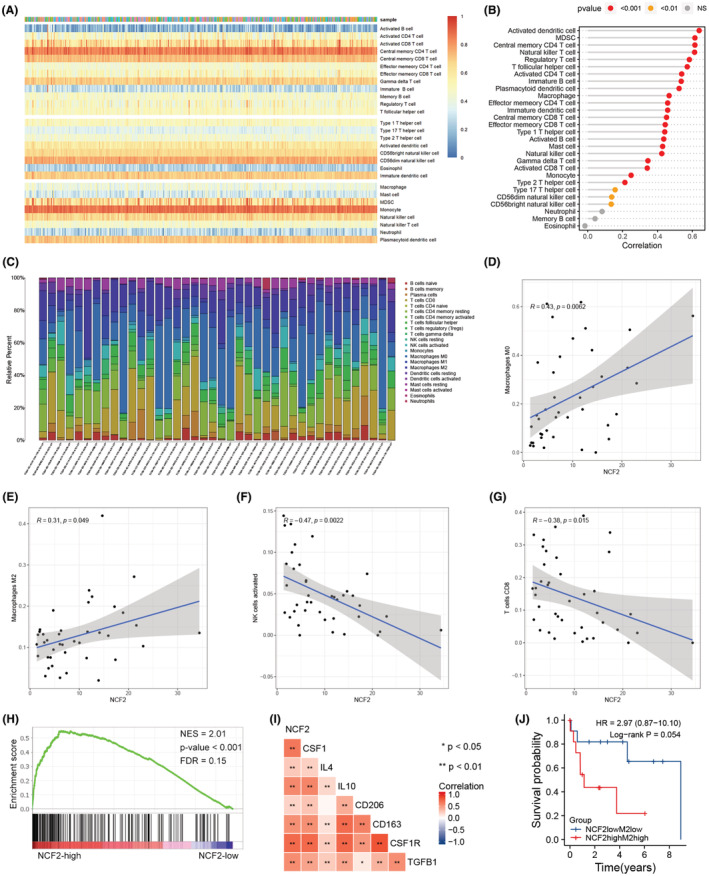
(A) A heatmap showing the immune cells infiltration assessed by ssGSEA algorithm. (B) The correlation between NCF2 expression level and each types of immune cells infiltrating in HCC tissue based on ssGSEA result. (C) A bar plot showing the immune cells infiltration assessed by CIBERSORT algorithm. (D) Positive correlation between M0 macrophages and NCF2 expression based on CIBERSORT result. (E) Positive correlation between M2 macrophages and NCF2 expression based on CIBERSORT result. (F) Negative correlation between activated NK cells and NCF2 expression based on CIBERSORT result. (G) Negative correlation between CD8+ T cells and NCF2 expression based on CIBERSORT result. (H) GSEA of chemokine signaling pathway induced by higher NCF2 expression level. (I) Correlation among NCF2, CSF1, IL4, IL10, CD206, CD163, CSF1R and TGFβ1 expression level. (J) Survival analysis of different NCF2 expression and M2 macrophages infiltration groups.

Considering that NCF2 was one of the DEGs between high‐ and low‐score group, we thus speculated that NCF2 might have effect on immune infiltration of HCC. We firstly demonstrated the infiltration of some categories of immune cells in the TME of the TCGA cohort by ssGSEA analysis (Figure 6A). The correlation analysis showed the relevance between the expression level of NCF2 and immune cells infiltration (Figure 6B). Then, to confirm the previous results, we used CIBERSORT algorithm to reassess the infiltration of immune cells of the TCGA cohort, and the assessment of 40 samples was reliable (*p* < 0.05, Figure 6C). The correlation analysis revealed that the increase in infiltration of M0 and M2 macrophages paralleled the rise in NCF2 expression level (Figure 6D,E), and the decrease in infiltration of activated NK cells and CD8+ T cells paralleled the rise in NCF2 expression level (Figure 6F,G). For further validation, we evaluated the immune cells infiltration of 232 HCC samples from ICGC database with CIBERSORT algorithm (Figure S3A), and explored the correlation between NCF2 expression level and immune cells infiltration, it showed that M0 and M2 macrophages were positively correlated with NCF2 expression (Figure S3B,C), and M1 macrophages was negatively correlated with NCF2 expression (Figure S3D).

Noteworthily, both two algorithms presented a significant correlation between the infiltration of macrophages and NCF2 expression level. Therefore, we conducted GSEA to explore the underlying mechanisms. The result showed that many immune‐related pathways (Figure S3E), including chemokine signaling pathway (Figure 6H), were enriched with higher expression level of NCF2. Colony Stimulating Factor (CSF) 1, interleukin (IL) 4 and IL10 are chemokines that promote the transformation of M0 macrophages into M2 macrophages, and CD206, CD163, CSF1R and Transforming Growth Factor (TGF) β1 are markers of M2 macrophages, for which we analyzed the correlation between their and NCF2 expression. As expected, the expression level of CFS1, IL4, IL10, CD206, CD163, CSF1R and TGFβ1 were strongly and positively correlated with NCF2 expression level (Figure 6I), and the tendencies of their expression were similar to that of NCF2 (Figure S3F). Finally, we divided the TCGA cohort into two groups according to the M2 macrophages abundance in CIBERSORT results and NCF2 expression level for survival analysis. Both NCF2^low^M2^low^ and MCF2^high^M2^high^ groups contained 12 samples respectively. The survival analysis showed that NCF2^low^M2^low^ group potentially had higher OS rate than NCF2^high^M2^high^ group (Figure 6J, *p* = 0.054, HR = 2.97, 95%CI 0.87–10.10).

## 4 DISCUSSION

NCF2 is a cytoplasmic component of NADPH oxidase,^19^ and it engages in the production of reactive oxygen species (ROS). NCF2 has been proved to play an important role in the microbial phagocytosis, and its mutation can cause chronic granulomatous disease.^20^, ^21^ Hence, NCF2 may function as an immune molecule to regulate anti‐tumor immunity and influence the prognosis of cancer patients. The role of NCF2 in the prognosis of cancer patients has previously been investigated by researchers. In lung cancer, the single nucleotide polymorphism of NCF2 was related to the prognosis.^22^ In gastrointestinal cancer, high expression level of NCF2 was associated with the poor prognosis of esophageal squamous cell carcinoma,^23^ and Zhang et al. revealed that upregulation of NCF2 promoted the angiogenesis and metastasis of gastric cancer through NF‐κB pathway.^24^ Besides, NCF2 was selected to construct a Macrophage‐Related Gene Prognostic Index for glioblastoma,^25^ and methylation of CpG sites of NCF2 was associated with the risk of breast cancer.^26^

However, the effect of NCF2 on the prognosis of HCC has not been explored before. In present study, we explored the role of NCF2 in the prognosis of HCC patients. From the results obtained so far, it seemed that NCF2 was an independent risk factor for HCC patients, and it could influence RFS and OS. Thereby our study presented some cues for forward studies.

ROS, including H_2_O_2_, superoxide anion and hydroxyl radical, can induce the oxidation of cellular components, like lipid, proteins and DNA. The consistent generation of ROS can trigger somatic mutation and cancerous transformation. Besides, ROS is an upstream molecule in NF‐κB signaling pathway, through which it can promote the proliferation of cancer cells. Interestingly, NCF2 was identified as a target gene of p53, with response elements of p53 in the promotor region.^27^ Overexpression of p53 induced the expression of NCF2, and p53 could inhibit apoptosis via p53 ‐ NCF2 ‐ ROS axis in HCT116 and HaCat cells.^27^ Recently, Kim J. et al confirmed the mutation of p53 was rarely observed in HCC, and wild‐type p53 could promote the proliferation of HCC cells by regulating metabolism with the participation of PUMA.^28^ All these imply wild‐type p53 might contribute to the progression of HCC by altering the metabolism of cancer cells via upregulating NCF2 expression.

Apart from the mechanisms mentioned above, NCF2 could also impact the outcome of HCC patients by regulating anti‐tumor immunity. In our study, the infiltration of M2 macrophages in the TME of HCC was positively associated with expression level of NCF2. Furthermore, NCF2 had strong and positive relationship with CSF1, IL4, IL10, CD206, CD163, CSF1R and TGFβ1, which indicates that NCF2 probably impact the RFS rate and OS rate by participating in the polarization of macrophages in the TME of HCC. Zhang et al.^29^ found that NOX4‐induced ROS could stimulate the production of various cytokines, including CSF1, CCL7 and IL8, via PI3K/Akt signaling pathway in non‐small cell lung cancer. And these cytokines can help with the polarization of stromal macrophages into M2 macrophages. Similar to NOX4, NCF2 is also involved in the generation of ROS when joins to form a NADPH‐oxidase complex. So, the mechanism mentioned above probably exist in HCC as well. Some studies showed that ROS promoted the polarization of TAMs to M2 macrophage through ROS/ERK and mTOR signaling pathway.^30^, ^31^ Interestingly, these pathways can regulate gene expression and protein synthesis, in which way various chemokines and cytokines may be produced for the chemotaxis and polarization of TAMs. NF‐κB signaling pathway is another probable mechanism in the regulation of macrophage polarization, for its activation can promote the production of some inflammatory cytokines. All these results may serve as references for further exploration on the function of NCF2.

Macrophages are rich in liver, and mainly divided into two types ‐ Kupffer cells and monocyte‐derived macrophages. TAMs play an important role in tumor‐associated inflammation, and can be polarized to distinct phenotype, including M1 and M2 macrophages. It is generally accepted that M1 macrophages exhibit anti‐tumor functions, while M2 macrophages are engaged in pro‐tumor process.^32^ IFN‐γ and LPS have been proved to induce the polarization of TAMs to M1 macrophages, while IL4, CFS1 and IL10 can mediate the polarization of TAMs to M2 macrophages.^33^, ^34^ It has been reported that M2 macrophages could inhibited CD8+ T cells and NK cells, thus accelerating the malignant progression and bringing worse prognosis.^35^, ^36^ Additionally, cytokine produced by M2 macrophages could also directly act on HCC cells to transform them into cancer stem cells through IL16/STAT3 signaling pathway, and promote their migration by CCL22‐CCR4 interaction.^37^, ^38^ In the last part of our study, we found that NCF2^high^M2^high^ group potentially showed lower OS rate, which illustrated the relationship between NCF2 expression and M2 macrophages infiltration from another prospective, and implied the role of NCF2 in the regulation of anti‐tumor immunity. We believe that conducting further experimental studies will bring intriguing results and provides broad mind for immunotherapy.

There were previously some studies that explored the relationship between TME and HCC with online database. LOXL2 was proved to be associated with the prognosis of HCC via regulating immune cell infiltration,^39^ and even cuproptosis‐related lncRNAs were corelated with immune response in HCC.^40^ In actuality, the TME was affected by diverse factors that are synergetic or antergic. To exploit new factors influencing the TME will help to reveal anti‐tumor immunity and to explore novel therapy.

In the present study, another two genes, ITGB6 and PLAUR, were also screened out as immune‐related genes. However, few studies have revealed their roles in HCC. In another study,^41^ ITGB6 was identified as an immune‐related gene by mining TCGA dataset. PLAUR, also known as UPAR, plays an important role in the invasion and metastasis role in HCC,^42^ and could influence the immune infiltration of HCC.^43^ Therefore, notwithstanding the result that ITGB6 and PLAUR were not independent prognostic factors in HCC, their effect in the progression of HCC is worth exploring.

This study still has some limitations. Firstly, our data were from TCGA database, and our clinical samples were collected from only one hospital, for which the biases of results are unavoidable. Secondly, we just implemented IHC to testify the prognostic role of NCF2 in HCC, while no experimental research was conducted to determine the mechanism by which NCF2 influences anti‐tumor immunity and the survival of HCC patients. Thirdly, although ssGSEA and CIBERSORT algorithms have been tested on reliability and are universally used, the real immune infiltration condition in HCC should be further affirmed. Overall, more studies are warranted to further explore the underlying mechanisms.

## 5 CONCLUSION

In summary, our research identified NCF2 as an independent prognostic factor for HCC patients, with its high expression predicting a poor prognosis. Besides, NCF2 could have effect on the infiltration of macrophages in the TME of HCC through some chemokines such as CSF1 and IL10. The present results may provide us with more precise methods for the prognosis of HCC patients and offer broad mind on the research of tumor immune microenvironment.

## AUTHOR CONTRIBUTIONS

**Ning Huang:** Formal analysis (equal); investigation (equal); visualization (lead); writing – original draft (lead). **Jing Zhang:** Data curation (lead); formal analysis (equal); validation (equal). **Shuwen Kuang:** Data curation (equal); formal analysis (equal). **Zhuo Li:** Investigation (equal); validation (equal). **Hong Zhao:** Writing – review and editing (equal). **Jianxiong Wu:** Writing – review and editing (equal). **Mei Liu:** Conceptualization (lead); funding acquisition (equal); resources (equal); supervision (equal). **Liming Wang:** Conceptualization (equal); funding acquisition (equal); resources (equal); supervision (lead).

## FUNDING INFORMATION

This work was supported by the CSCO‐Bayer Research Foundation of Cancer (Y‐bayer202001‐0087), the National Natural Science Foundation (81641113, 81972767), and the Chinese Academy of Medical Sciences (CAMS) Innovation Fund for Medical Sciences (CIFMS) (2021‐I2M‐1‐018, 2021‐I2M‐1‐066), PR China. The funders had no role in study design, data collection and analysis, interpretation of data, or preparation of the manuscript.

## CONFLICT OF INTEREST

The authors declare that they have no conflict of interests.

## ETHICS APPROVAL AND CONSENT TO PARTICIPATE

The study protocol was approved by the Independent Ethics Committee of Cancer Hospital Chinese Academy of Medical Sciences. All patients provided written informed consent in accordance with the Declaration of Helsinki.

## Supporting information


Data S1.

## Data Availability

Due to the nature of this research, the clinical data that support the findings of this study are available from the corresponding authors, Liming Wang, upon reasonable request. The data from TCGA can be found on https://cancergenome.nih.gov/, and that from ICGC can be found on https://dcc.icgc.org/.
