# Supplementary material for: Role of NCF2 as a potential prognostic factor and immune infiltration indicator in hepatocellular carcinoma
Source: Cancer Med. 2023 Jan 20;12(7):8991–9004. doi: 10.1002/cam4.5597 (PMC10134316; doi:10.1002/cam4.5597)
Supplement: Supplementary file 1 — Data S1. [file CAM4-12-8991-s001.zip › CAM4_5597_supplementary data-submission.docx]

**Supplementary Data**

**Supplementary Table 1 The Clinicopathological Characteristics of CHCAMS Cohort**

| **Clinicopathological Characteristics** | **Cases (n, %)** |
| --- | --- |
| Gender |  |
| female | 7 (21.9%) |
| male | 25 (78.1%) |
| Age |  |
| ≤60 | 23 (71.9%) |
| >60 | 9 (28.1%) |
| Cirrhosis |  |
| no | 6 (6.3%) |
| yes | 26 (81.3%) |
| Lymphovascular Space Invasion (LVSI) |  |
| no | 20 (62.5%) |
| yes | 12 (37.5%) |
| Capsular Invasion (CapI) |  |
| no | 12 (37.5%) |
| yes | 20 (62.5%) |
| Tumor size |  |
| ≤5cm | 19(59.4%) |
| >5cm | 13(40.6) |
| Necrosis |  |
| no | 20 (62.5%) |
| yes | 10 (31.3%) |
| unclear | 2 (6.2%) |
| Differentiation |  |
| Well | 0(0%) |
| moderately | 22(68.8%) |
| poorly | 10(31.2%) |
| Hepatitis^(1)^ |  |
| 0 | 1 (3.1%) |
| 1 | 4 (12.5%) |
| 2 | 23 (71.9%) |
| 1+Hepatitis C | 3 (9.4%) |
| unclear | 1 (3.1%) |
| Postoperative treatment |  |
| radiotherapy | 8(25.0%) |
| interventional therapy | 6(18.8%) |
| no | 19(59.4%) |

1. 0 = all antigen and antibody(-) or only HBsAb(+), 2 = HBsAg(+), 1 = other cases

**Supplementary Table 2 The Clinicopathological Characteristics of TCGA Cohort**

**Supplementary Table 3 The Clinicopathological Characteristics of CHCAMS Cohort**

**Figures**


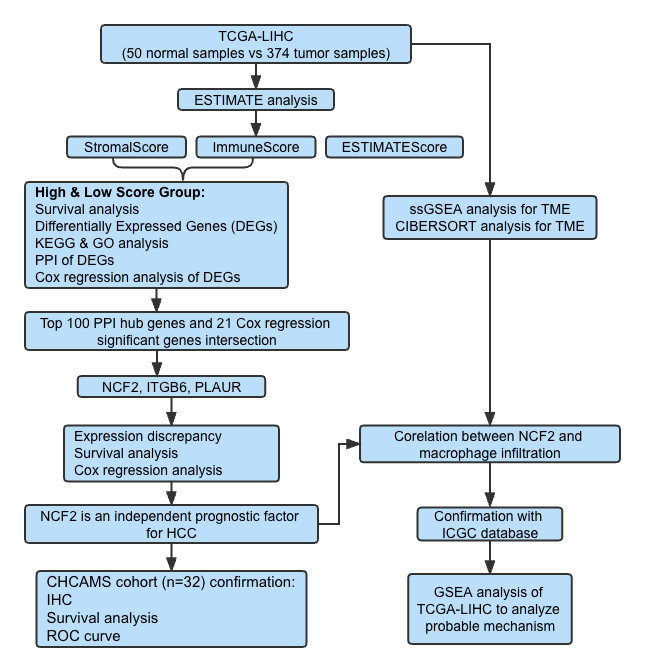


**Supplementary Figure 1** The flow chart of data collection and analysis


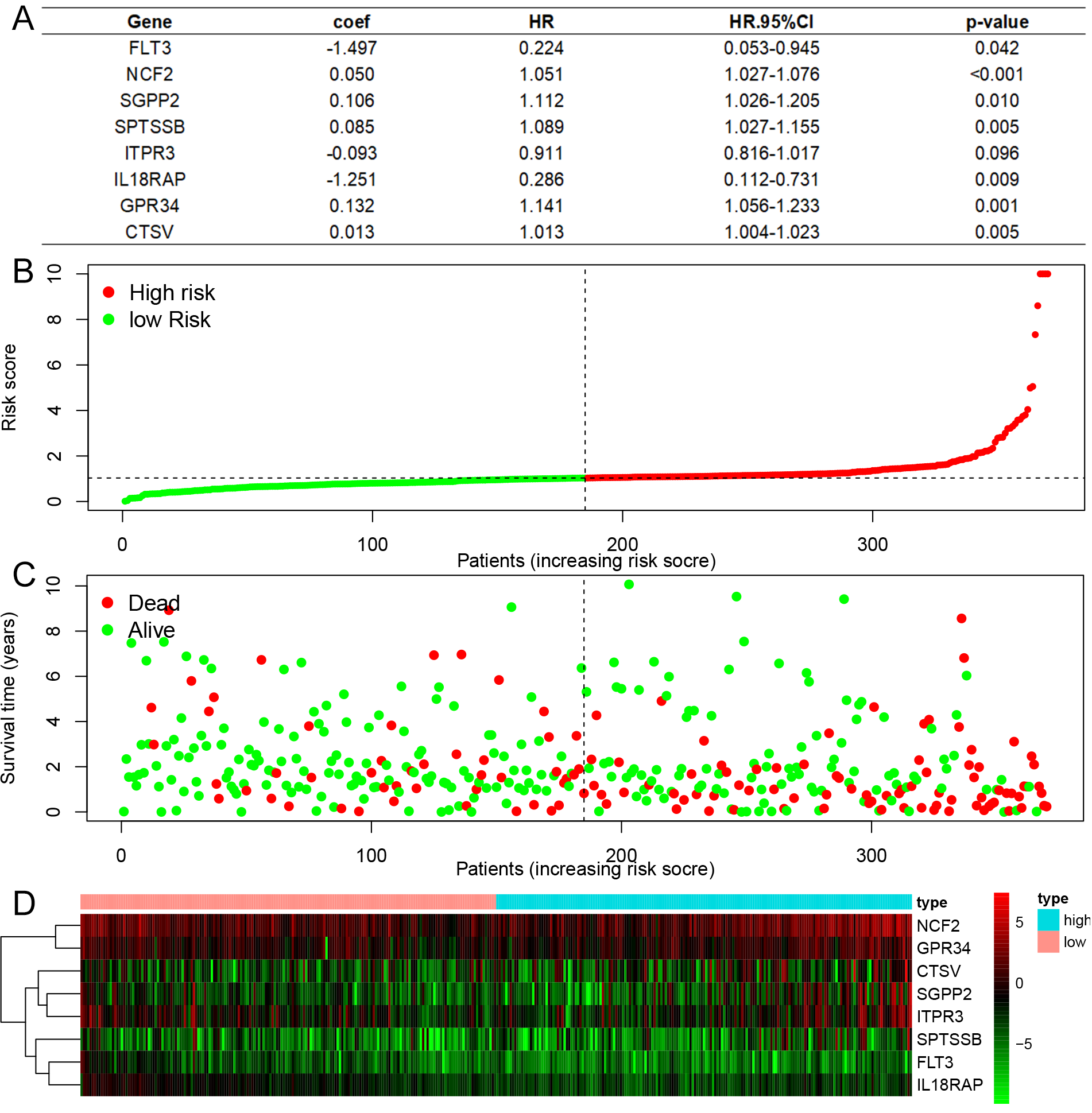


**Supplementary Figure 2** (A) Genes selected from prognosis and immune related genes for the construction of prognostic model. (B) Low and high riskScore group of patients in TCGA Cohort. (C) The prognostic discrepance between high and low riskScore group. (D) The expression heatmap of genes included in the prognostic model.


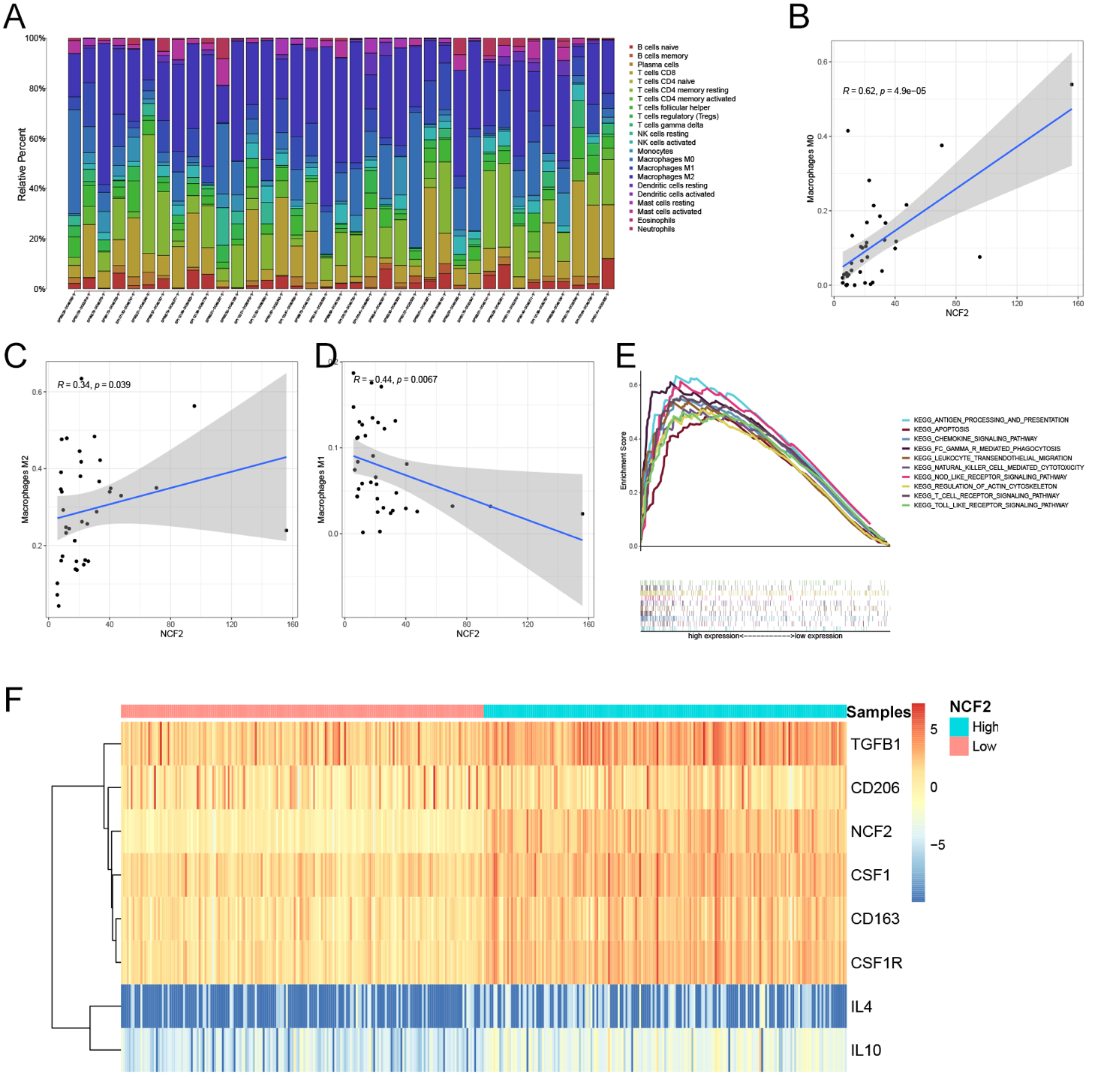


**Supplementary Figure 3** (A) Relative abundance of Immune cells infiltration in ICGC HCC samples. (B-D) The correlation between NCF2 expression level and different macrophages. (E) Result of GSEA analysis of TCGA cohort. (F) A heatmap showing M2 macrophages-related molecules expression tendency in low- and high-NCF2 groups.
